# The Impact of Paeoniflorin on *α*-Synuclein Degradation Pathway

**DOI:** 10.1155/2015/182495

**Published:** 2015-11-26

**Authors:** Zenglin Cai, Xinzhi Zhang, Yongjin Zhang, Xiuming Li, Jing Xu, Xiaomin Li

**Affiliations:** ^1^Lianyungang First People's Hospital Postdoctoral Innovative Practice Base, Nanjing Medical University Postdoctoral Research Station, Lianyungang 222002, China; ^2^Department of Neurology, Affiliated Lianyungang Hospital of Xuzhou Medical College, Lianyungang 222002, China; ^3^Department of Emergency, Affiliated Lianyungang Hospital of Xuzhou Medical College, Lianyungang 222002, China

## Abstract

Paeoniflorin (PF) is the major active ingredient in the traditional Chinese medicine Radix. It plays a neuroprotective role by regulating autophagy and the ubiquitin-proteasome degradation pathway. In this study, we found PF significantly reduced cell damage caused by MPP+, returning cells to normal state. Cell viability significantly improved after 24 h exposure to RAPA and PF in the MPP+ group (all *P* < 0.01). CAT and SOD activities were significantly decreased after PF and RAPA treatment, compared with MPP+ (*P* < 0.001). In addition, MPP+ activated both LC3-II and E1; RAPA increased LC3-II but inhibited E1. PF significantly upregulated both LC3-II (autophagy) and E1 (ubiquitin-proteasome pathway) expression (*P* < 0.001), promoted degradation of *α*-synuclein, and reduced cell damage. We show MPP+ enhanced immunofluorescence signal of intracellular *α*-synuclein and LC3. Fluorescence intensity of *α*-synuclein decreased after PF treatment. In conclusion, these data show PF reversed the decline of proteasome activity caused by MPP+ and significantly upregulated both autophagy and ubiquitin-proteasome pathways, promoted the degradation of *α*-synuclein, and reduced cell damage. These findings suggest PF is a potential therapeutic medicine for neurodegenerative diseases.

## 1. Introduction

Genetic, pathological, and biochemical studies show *α*-synuclein plays a role in Parkinson's disease (PD) and Lewy body associated disease. However, it is unclear how *α*-synuclein causes neurodegenerative disease [1]. The water-soluble oligomer of *α*-synuclein is toxic, destabilizing the cellular environment and injuring mitochondria [2]. In addition, *α*-synuclein readily forms oligomeric species, which are cytotoxic [2]. The ubiquitin-proteasome system and autophagy-lysosomal pathway are critical degradation pathways of *α*-synuclein and are closely correlated with the pathogenesis of PD.

Paeoniflorin (PF) is a monoterpene glycoside and a major active ingredient of the traditional Chinese medicine Radix. Recent studies show PF has neuroprotective effects via modulation of ROS production and apoptosis in the mitochondrial pathway in injured neurons in vitro [3]. PF also has neuroprotective effects on animal brain ischemia via inhibition of MAPKs/NF-kappaB mediated peripheral and cerebral inflammatory response [4, 5]. Our team previously showed that, in addition to inhibiting inflammation, PF also plays a neuroprotective role in a neuronal injury model by regulating autophagy [6]. However, mechanistic details are still lacking.

## 2. Materials and Methods

### 2.1. Cell Culture and Treatments

Rat pheochromocytoma (PC12) cell lines were a kind gift from the Institute of Neuroscience, Soochow University. All cell lines were maintained in RPMI-1640 medium supplemented with 10% heat-inactivated fetal bovine serum (Gibco, Langley, OK, USA). Cells were seeded in culture flasks, 24- or 96-well plates, to a confluence of 60–70%. Cells were treated for 24 hrs with MPP+ (Sigma-Aldrich, St. Louis, MO, USA) (0.5 mMol/L) in RPMI-1640 medium, Paeoniflorin (50 uM) (Sigma-Aldrich, St. Louis, MO, USA), and Rapamycin (0.2 *μ*g/mL) (Santa Cruz Biotechnology, Santa Cruz, CA, USA). Proteasome activity (Promega, Madison, WI, USA), catalase (CAT) activity, and superoxide dismutase (SOD) activity (Cayman Chemical, Ann Arbor, Michigan, USA) were measured with the appropriate assay kits according to the manufacturer's protocols.

### 2.2. MTT Assay for Cell Viability

Cells were cultured in 96-well plates with 10% calf serum RPMI-1640 medium at a density of 1 × 10^5^/mL cells/well (200 *μ*L/well). Cells were treated in exponential growth phase with MPP+, Paeoniflorin, or Rapamycin and then incubated for 24 h. The culture medium was refreshed and 20 *μ*L MTT solution (final concentration: 0.5 mg/mL) (Beyotime Institute of Biotechnology, Jiangsu, China) was added to each well. Cells were incubated at 37°C for an additional 4 h in dark. After incubation, the medium with MTT was removed, and 150 *μ*L dimethyl sulfoxide (DMSO) was added to each well to dissolve the formazan dye crystals on a shaker for 15 min. Absorbance was measured at 492 nm. Results were calculated relative to controls, assuming an absorbance of 100%. All data are expressed as mean ± SD (*n* = 5).

### 2.3. Observation under Light Microscopy

Cells were plated, serum deprived, and treated with MPP+ (0.5 mMol/L) and Paeoniflorin (50 uM). Cell growth, cell shape, and adherent cells were observed under a light microscope at 100x and 400x optical microscope magnification 24 h after treatment.

### 2.4. Western Blotting

Western blot analysis was performed as previously described [7]. Cells were lysed and sonicated in SDS lysis buffer (Beyotime Institute of Biotechnology, Jiangsu, China). After protein separation by electrophoresis on 12% SDS polyacrylamide gels with Tris-glycine running buffer, samples were transferred onto a polyvinylidene difluoride (PVDF) membrane (Millipore, Bedford, MA, USA) followed by immunoblotting with antibodies overnight at 4°C with gentle agitation as follows: anti-*α*-synuclein (1 : 1000, Cell Signaling Technology, Danvers, MA, USA, 2642), anti-LC3 (1 : 1000, Abcam, Cambridge, UK, ab62721), anti-UBE1 (1 : 1000, Cell Signaling Technology, Danvers, MA, USA, 4891S), and *β*-actin (1 : 1000, Sigma-Aldrich, St. Louis, MO, USA, A3854). After four washing procedures in TBS containing 0.1% Tween 20, the membranes were incubated with horseradish peroxidase-conjugated secondary antibody (Beyotime Institute of Biotechnology, Jiangsu, China) for 2 h at room temperature. Each measurement was performed on five independent runs. The images were captured using the Odyssey Infrared Imaging System (LI-COR Biosciences, Lincoln, NE) and band intensities were calculated by the densitometric analysis using ImageJ software.

### 2.5. Immunofluorescence Microscopy

Details are discussed in previously published literature [7]. Briefly, PC12 cells were plated on noncoated 12 mm coverslips and treated with MPP+ (0.5 mMol/L, 24 h exposure) and Paeoniflorin (50 uM, 24 h) and then fixed in ice-cold 4% paraformaldehyde for 15 min. The cells were then exposed to primary anti-LC3 antibody (1 : 250, Abcam, Cambridge, UK) or anti-*α*-synuclein antibody (1 : 250, Abcam, Cambridge, UK) for 1.5 h at 37°C. After appropriate secondary antibody (1 : 500, Cy3-labeled Goat Anti-Rabbit for anti-LC3, FITC-labeled Goat Anti-Mouse IgG for anti-*α*-synuclein, Beyotime Institute of Biotechnology, Jiangsu, China) treatment for 1 h, the labeled cells were stained with 4′,6-diamidino-2-phenylindole (DAPI, 0.3 *μ*g/mL) for 15 min and evaluated by a Laser Scanning Confocal Microscope (Leica TCS SP2 CLSM). Images were collected and processed using the imaging software provided by the Leica TCS system.

### 2.6. Statistical Analysis

All experiments were performed in triplicate, and the results are presented as mean ± standard deviation (SD). Two group comparisons were performed using Student's* t*-test. Multiple group comparisons were performed using one-way analysis of variance and Fisher's least significant difference. Values of *P* < 0.05 were set as statistically significant.

## 3. Results

### 3.1. Morphological Changes under Ordinary Light Microscope

**Figure 1:**
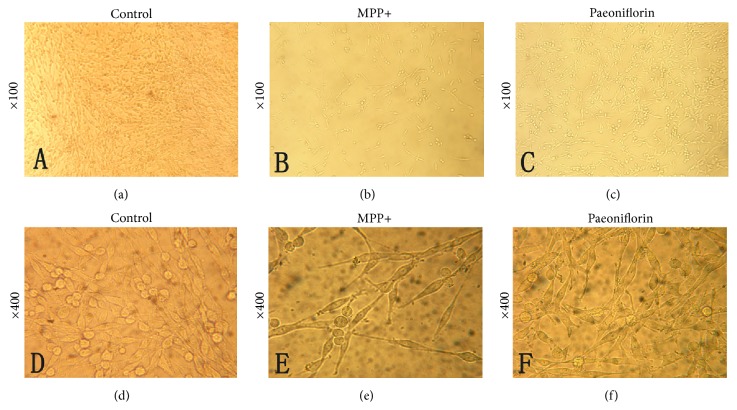
Paeoniflorin shows a protective effect from injury by MPP+ under light microscopy. (a) Normal PC12 cells (×100). (b) MPP+ treated PC12 cells at 24 h (×100-fold). (c) Paeoniflorin treated cells 24 h after MPP+ treatment (×100). (d) Normal PC12 cells (×400). (e) MPP+ 24 h (×400). (f) Paeoniflorin treated cells 24 h after MPP+ treatment (×400).

Cells were fibroblast-like, with long overshoot, and adherent under normal growth state (Figures 1(a) and 1(d)). After MPP+ treatment, cell number decreased and cell rounding was smaller. Cells became wrinkled, and we found cells in suspension (Figures 1(b) and 1(e)). Paeoniflorin significantly reduced the damage caused by MPP+, and cells returned to normal state (Figures 1(c) and 1(f)).

### 3.2. PF and RAPA Reversed the Cellular Damage Caused by MPP+

**Figure 2:**
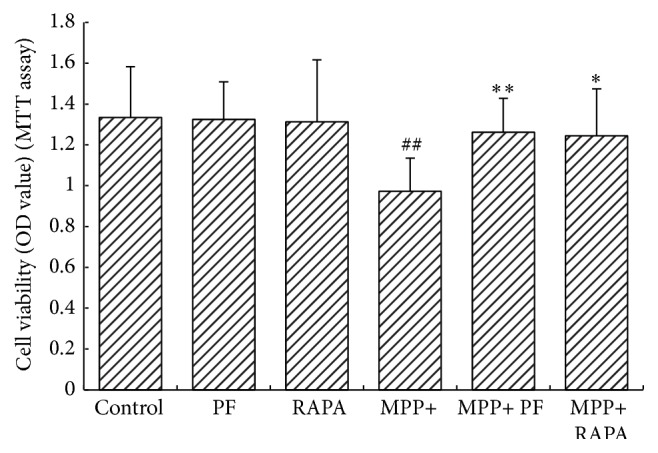
MTT cell viability assay. Both PF and Rapamycin treatment significantly improved cell viability in MPP+ treated group (^#^ *P* < 0.05, ^##^ *P* < 0.01 versus control group; ^*∗*^ *P* < 0.05, ^*∗∗*^ *P* < 0.01 versus corresponding control group; mean ± SD, *n* ≥ 6).

In order to assess the impact of PF and RAPA on PC12 cellular damage, we determined viability of PC12 cells 24 h after MTT treatment and then compared results after PF or RAPA treatment. Absorbance value (OD) was measured on a microplate reader. In the MPP+ group, we found RAPA and PF treatment significantly improved cell viability (*t* values = 5.988, 3.766, resp.; *P* values = 0.001, 0.009, resp.). We found no significant difference comparing RAPA with PF treatment groups (*P* > 0.05) (Figure 2). We found PF and RAPA did not significantly affect cell viability under normal growth state.

### 3.3. SOD/CAT Activity in PC12 Cells

**Figure 3:**
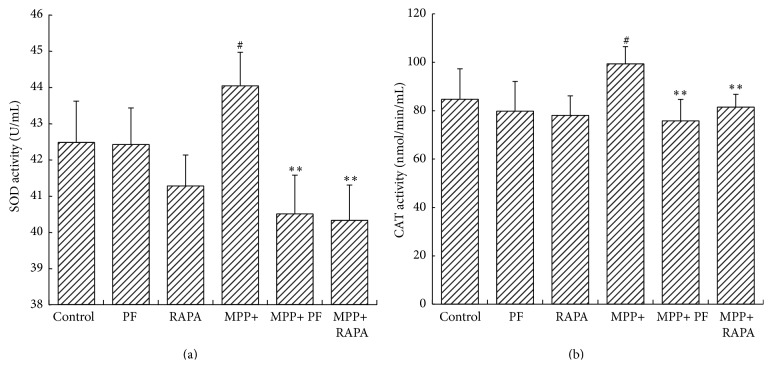
Superoxide dismutase (SOD) and catalase (CAT) activity in cells. (a) Rapamycin and PF significantly decreased SOD activity after MPP+ treatment. (b) CAT activity decreased in MPP+ group (^#^ *P* < 0.05, ^##^ *P* < 0.01 versus control group; ^*∗*^ *P* < 0.05, ^*∗∗*^ *P* < 0.01 versus corresponding control group, mean ± SD, *n* ≥ 6).

To investigate the protective effect of PF and RAPA on cell damage caused by MPP+, we examined SOD and CAT activity in PC12 cells. In normal growth conditions, PF and RAPA did not influence SOD or CAT activity. We found CAT activity increased in cells exposed to MPP+ compared with controls (84.73 ± 12.61 versus 99.33 ± 7.14 nmol/min/mL; *P* = 0.035). After PF and RAPA treatment, CAT activity decreased compared with MPP+ group (PF: 75.77 ± 8.89 versus 99.33 ± 7.14 nmol/min/mL; *P* = 0.002) (RAPA: 81.45 ± 5.32 versus 99.33 ± 7.14 nmol/min/mL; *P* < 0.001). SOD activity decreased compared with MPP+ group as well (PF: 40.50 ± 1.07 versus 44.04 ± 0.92 nmol/min/mL; *P* < 0.001; RAPA: 40.33 ± 0.97 versus 44.04 ± 0.92 nmol/min/mL; *P* < 0.001) (Figure 3).

### 3.4. Changes in Proteasome Activity following MPP+ and PF Treatment

**Figure 4:**
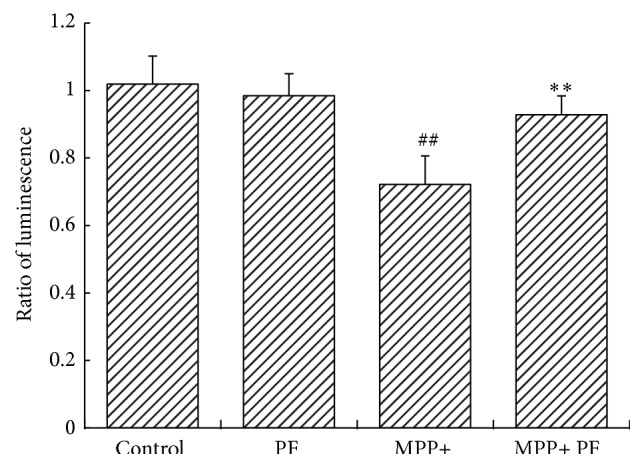
Change in proteasome activity following MPP+ and PF treatment. Under normal circumstances, the impact of PF on proteasome activity was not statistically significant (*P* = 0.076). MPP+ inhibited the ubiquitin-proteasome activity (*P* = 0.002). Paeoniflorin reversed the decline of proteasome activity caused by MPP+ (*P* = 0.004) (^##^ *P* < 0.01 MPP+ group versus control group; ^*∗∗*^ *P* < 0.01 versus MPP+ group; mean ± SD, *n* ≥ 6).

To measure the impact of PF on proteasome activity, we used proteasome activity kit to detect changes after MPP+ and PF treatment. Under normal conditions, PF had no significant effect on proteasome activity. However, MPP+ significantly inhibited the ubiquitin-proteasome pathway (*P* = 0.002). PF ameliorated the decline in proteasome activity caused by MPP+ (*P* = 0.004) (Figure 4).

### 3.5. PF Promotes *α*-Synuclein Degradation through Both Autophagy and the Ubiquitin-Proteasome Pathway

**Figure 5:**
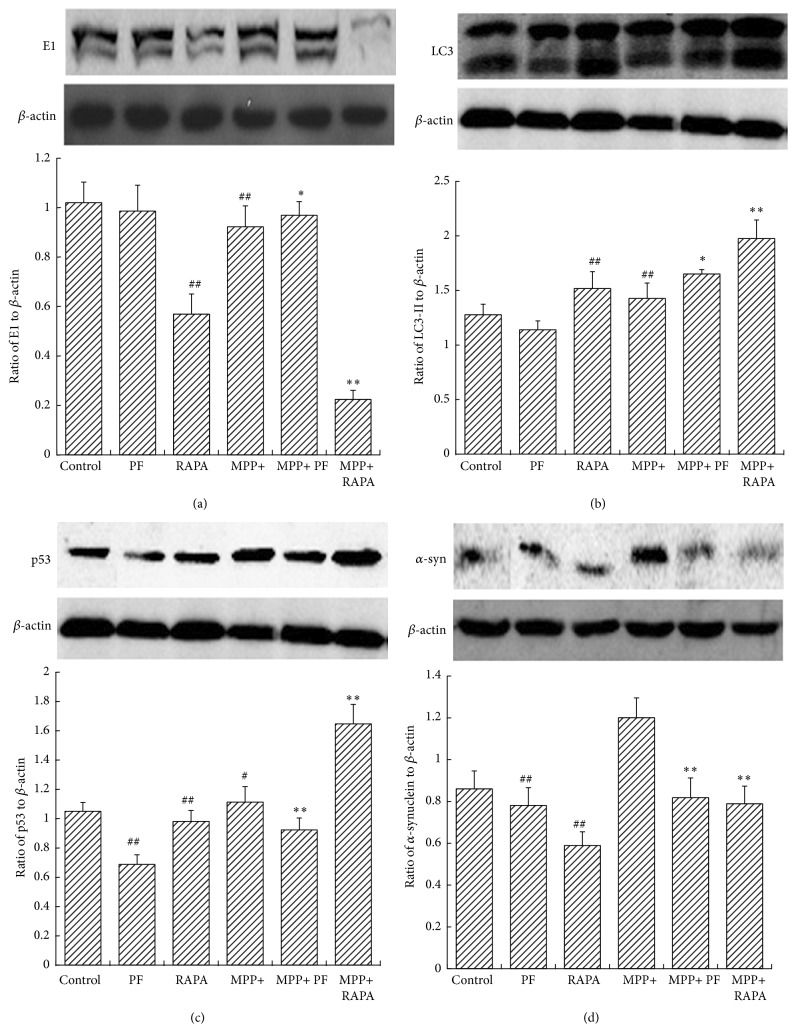
Rapamycin and PF impact on *α*-synuclein and its degradation pathway. Western blots (upper panel in (a), (b), (c), and (d)) and statistical analysis of optical density measurements (lower panel in (a), (b), (c), and (d)) in PC12 cells after treatment with MPP+, PF, and RAPA for (a) E1, (b) LC3-II, (c) p53, and (d) *α*-synuclein. In MPP+ group, RAPA increased LC3-II and inhibited E1. PF upregulated both LC3-II and E1 significantly. Values represent mean ± SEM (*n* = 5). ^#^ *P* < 0.05, ^##^ *P* < 0.01 versus control group. ^*∗*^ *P* < 0.05, ^*∗∗*^ *P* < 0.01 versus corresponding control group.

Under normal growth conditions, PF has no impact on autophagy or the ubiquitin-proteasome pathway. In contrast, RAPA induces autophagy and inhibits the ubiquitin-proteasome pathway in normal conditions. MPP+ activates the autophagy pathway but does not affect degradation of aggregation-prone *α*-synuclein. We show, in the MPP+ group, RAPA increased autophagy and inhibited E1 from promoting autophagic degradation of *α*-synuclein. We found PF significantly upregulated both pathways, promoted the degradation of *α*-synuclein, and reduced cell damage. PF and RAPA treatment decreased p53 levels in both control and MPP+ group (Figure 5). These data suggest that PF can simultaneously improve the function of ALP and UPS to facilitate the degradation of *α*-synuclein, but not by affecting the function of p53.

### 3.6. Fluorescence Results

**Figure 6:**
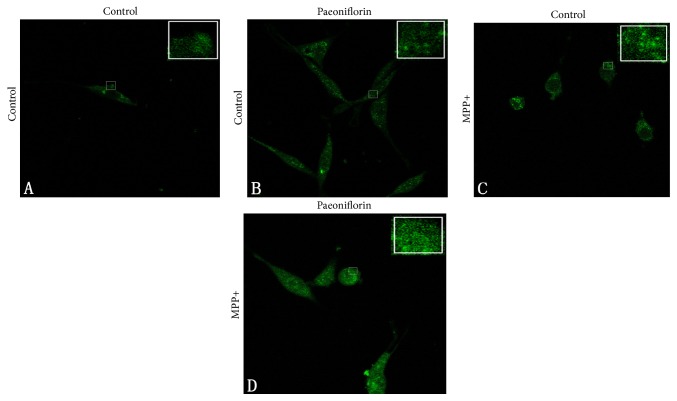
Paeoniflorin and MPP+ treatment on *α*-synuclein aggregation: (A) normal condition; (B) Paeoniflorin treatment did not affect *α*-synuclein aggregation; (C) after MPP+ treatment, *α*-synuclein tends to accumulate and aggregates appeared in the cytoplasm; (D) but after MPP+ and then Paeoniflorin treatment, cytoplasmic *α*-synuclein aggregates significantly reduced.

**Figure 7:**
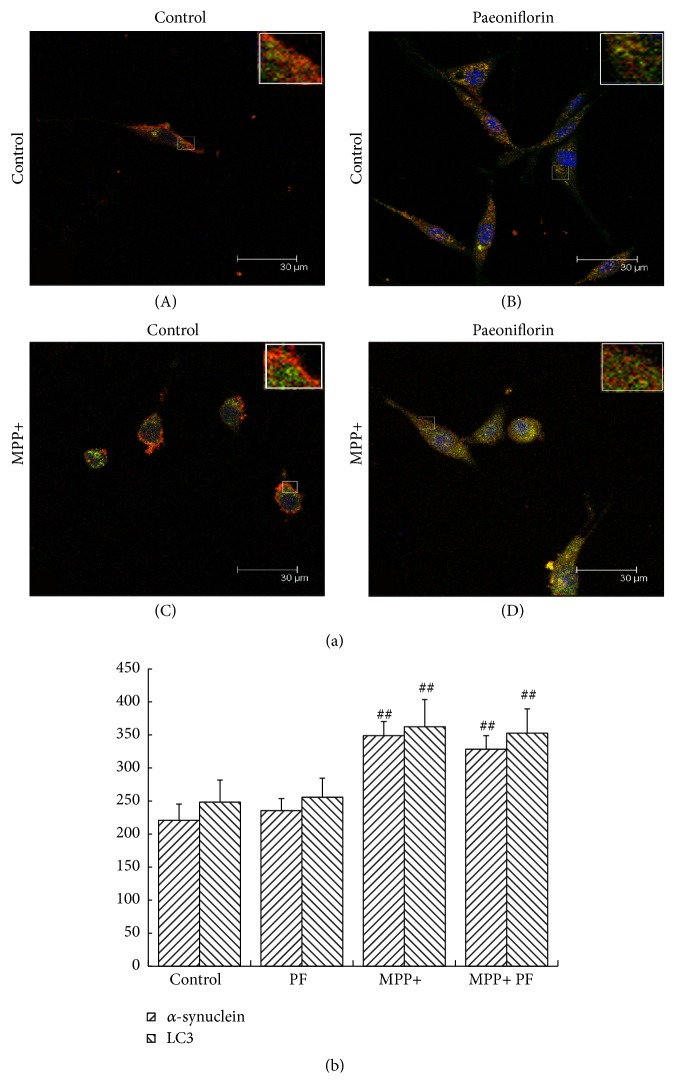
*α*-synuclein and LC3 colocalization in PC12 cells. Immunostain of *α*-synuclein (green) and LC3 (red) (a) and statistical analysis of optical density measurements (b), under normal conditions (A) and Paeoniflorin treatment (B), did not affect *α*-synuclein and LC3 and their colocalization; after MPP+ treatment (C), the signals of *α*-synuclein and LC3 were increased and lost their colocalization (more obvious in the enlarged figures). LC3 mainly aggregated in the peripheral cytoplasm but *α*-synuclein was distributed throughout the cell body. After MPP+ treatment and Paeoniflorin (D), the signals of *α*-synuclein and LC3 were both decreased and colocalization remained evident. Scale bar: 30 um. Quantification of immunostain results showed Paeoniflorin treatment did not affect fluorescence intensity of *α*-synuclein and LC3 in normal growth state (*P* > 0.05) but decreased the fluorescence intensity of *α*-synuclein and LC3 in MPP+ group. Values represent mean ± SEM (*n* = 5). ^#^ *P* < 0.05, ^##^ *P* < 0.01 versus control group.

We investigated the effect of PF on *α*-synuclein aggregation and autophagy pathway by observing the colocalization of *α*-synuclein and LC3 in cells treated with PF and MPP+ using laser confocal microscopy. We found under normal conditions PF had no significant effect on the fluorescence intensity of *α*-synuclein (Figure 6(B)) or LC3 (Figure 7(B)). Consistent with our previous findings, MPP+ enhanced the immunofluorescence signal of intracellular *α*-synuclein and LC3, and cytosolic aggregates of *α*-synuclein appeared (Figure 6(C)); *α*-synuclein and LC3 colocalization was reduced. After PF treatment, the fluorescence intensity of *α*-synuclein and LC3 decreased, but colocalization was still evident (Figure 7(D)).

## 4. Discussion

*α*-synuclein aggregation is a characteristic of many neurodegenerative diseases including Parkinson's disease (PD), Lewy bodies (LBs) dementia, and *α*-synuclein pathology diseases [8]. Mounting evidence shows that *α*-synuclein misfolding, aggregation, and abnormal degradation cause dopaminergic neuron death. This in turn triggers PD pathogenesis and has an important role in the disease development process [2, 9]. The ubiquitin-proteasome and autophagy-lysosomal pathways are two main paths that clear proteins and organelles in eukaryotic cells. The proteasome is a barrel shaped multiprotein complex that degrades short-lived nuclear and cytosolic proteins [10]. Proteasome substrates are forced to linearize and travel through a narrow cylindrical pore proteasome, preventing the clearance of oligomers and aggregates [11, 12].

PF offers multiple neuroprotective benefits including improving brain blood circulation, supporting anti-inflammation, attenuating dopaminergic neurotoxicity, and alleviating symptoms of degenerative diseases [4, 13]. Most recently, studies have focused on the role of PF in protein aggregation diseases such as Parkinson's disease and Alzheimer's disease and in the degradation of aggregation-prone proteins. For example, Chang et al. [14] found that PF significantly prohibited the aggregation of polyQ proteins and upregulated HSF1 and HSP70 chaperones in both 293 ATXN3/Q75-GFP cells and SH-SY5Y ATXN3/Q75-GFP cells. Others have shown that PF protects PC12 cells against MPP+ induced injury by upregulating the autophagic pathway [15].

In this study, we show PF and RAPA reduced CAT and SOD activities, thereby protecting cells by avoiding oxidative stress injury caused by MPP+. RAPA and PF increased cell viability in MPP+ cells. PF significantly reduced the damage induced by MPP+, and the cells returned to normal state. Under normal growth state, PF had no statistically significant impact on autophagy or the ubiquitin-proteasome pathway. Further, we found, after MPP+ treatment, RAPA increased autophagy promoting autophagic degradation of *α*-synuclein. PF significantly upregulated both autophagy and ubiquitin-proteasome pathways, promoted the degradation of *α*-synuclein, and reduced cell damage. We argue that the PF impact on ALP and UPS is not mediated via p53 function.

p53 plays a significant role in the regulation of autophagy, degradation, and recycling of macromolecules and organelles, especially in nutrient deprived conditions [16, 17]. p53 maintains autophagy homeostasis and regulates autophagy flux by increasing cell activity [18]. Rapamycin treatment causes proteasome-dependent degradation of p53 [19, 20] in the cytoplasm and nucleus and increases autophagy by inhibiting mTOR. Our findings show PF protected against MPP+ induced injury in PC12 cells, suggesting p53 is involved in this process. We argue that PF does not regulate autophagy through mTOR, but rather by other signaling pathways.

## 5. Conclusion

Our findings show PF reduced CAT and SOD activities, increased cell viability, and protected cells against oxidative stress caused by MPP+. In addition, PF significantly reduced the damage caused by MPP+, returning cells to normal state. Moreover, PF upregulated both autophagy and ubiquitin-proteasome pathways in a p53 independent function. Finally, PF promoted the degradation of *α*-synuclein and reduced cell damage. Thus, we suggest PF is a potential therapeutic medicine for neurodegenerative diseases.
